# Identification of a Novel Linear B-Cell Epitope on the Nucleocapsid Protein of Porcine Deltacoronavirus

**DOI:** 10.3390/ijms21020648

**Published:** 2020-01-19

**Authors:** Jiayu Fu, Rui Chen, Jingfei Hu, Huan Qu, Yujia Zhao, Sanjie Cao, Xintian Wen, Yiping Wen, Rui Wu, Qin Zhao, Xiaoping Ma, Xiaobo Huang

**Affiliations:** 1 Research Center of Swine Disease, College of Veterinary Medicine, Sichuan Agricultural University, Chengdu 611130, China; 2 Sichuan Science-observation Experimental station of Veterinary Drugs and Veterinary Diagnostic Technology, Ministry of Agriculture, Chengdu 611130, China; 3 National Teaching and Experiment Center of Animal, Sichuan Agricultural University, Chengdu 611130, China

**Keywords:** porcine deltacoronavirus, nucleocapsid, monoclonal antibodies

## Abstract

Porcine deltacoronavirus (PDCoV), first identified in 2012, is a swine enteropathogen now found in many countries. The nucleocapsid (N) protein, a core component of PDCoV, is essential for virus replication and is a significant candidate in the development of diagnostics for PDCoV. In this study, monoclonal antibodies (mAbs) were generated and tested for reactivity with three truncations of the full protein (N1, N2, N3) that contained partial overlaps; of the five monoclonals chosen tested, each reacted with only the N3 truncation. The antibody designated 4E88 had highest binding affinity with the N protein and was chosen for in-depth examination. The 4E88 epitope was located to amino acids 308-AKPKQQKKPKK-318 by testing the 4E88 monoclonal for reactivity with a series of N3 truncations, then the minimal epitope, 309-KPKQQKKPK-317 (designated EP-4E88), was pinpointed by testing the 4E88 monoclonal for reactivity with a series of synthetic peptides of this region. Homology analysis showed that the EP-4E88 sequence is highly conserved among PDCoV strains, and also shares high similarity with sparrow coronavirus (HKU17), Asian leopard cat coronavirus (ALCCoV), quail coronavirus (UAE-HKU30), and sparrow deltacoronavirus (SpDCoV). Of note, the PDCoV EP-4E88 sequence shared very low similarity (<22.2%) with other porcine coronaviruses (PEDV, TGEV, PRCV, SADS-CoV, PHEV), demonstrating that it is an epitope that can be used for distinguishing PDCoV and other porcine coronavirus. 3D structural analysis revealed that amino acids of EP-4E88 were in close proximity and may be exposed on the surface of the N protein.

## 1. Introduction

The genus *Deltacoronavirus* is a relatively new member of the Coronavirus subfamily, that consists of avian and mammalian CoVs [1]. Among these is porcine deltacoronavirus (PDCoV), originally discovered from fecal samples of pigs in Hong Kong in 2012 [2]. Since then, PDCoV has been reported in multiple states of the United States and Canada [3,4,5,6], South Korea [7], mainland China [8,9] and Thailand [10] causing economic losses to each country’s swine industry. Clinically, Porcine deltacoronavirus (PDCoV) is indistinguishable from porcine epidemic diarrhea virus (PEDV) and transmissible gastroenteritis virus (TGEV), both Alphacoronaviruses, it is characterized by severe diarrhea, vomiting, and dehydration in piglets, and histopathological lesions typical of atrophic enteritis [11]. The clinical and epidemiological similarities between PDCoV and other porcine intestinal pathogenic coronaviruses make diagnosis and treatment of these viruses a challenge, highlighting the need for discriminating diagnostic methods [12].

PDCoV is an enveloped, single-stranded, positive-sense RNA virus with a 25 kb genome [13]. In the genome opening reading frames(ORFs), ORF1a and ORF1b account for two-thirds of its genome, which encode two polymerase proteins, pp1a and pp1ab [14]. The last one-third of the genome encodes four structural proteins: spike (S protein), envelope (E protein), membrane (M protein), nucleocapsid (N protein), and three accessory proteins (NS6 and NS7/NS7a) [15,16]. NS7 ORF is included into N gene sequence. Moreover, NS7a is contained into NS7 ORF [16]. The N protein is a phosphoprotein and binds to RNA genome, which supplies a structural basis to the helical nucleocapsid [17,18]. The common characteristics for all CoVs N proteins are high expression levels early in the infection and high anti-N antibody levels. N protein also owns multiple functions in pathogenesis, viral replication, and immune system interference [17]. These characteristics make the N protein an ideal target for development of serological methods based on purified protein [19] or antigenic epitopes [20].

PDCoV N protein is highly conserved among PDCoV strains but had low sequence identity with other porcine coronavirus, such as PEDV, TGEV, and PRCV [21]. Although CoV N proteins have low sequence identity, all share the same domain and structure organization [18,22]. For diagnosis of PDCoV, serological assays based on N protein, such as indirect ELISA and fluorescent microsphere immunoassay, have proven to be highly sensitive [23]. Monoclonal antibodies of PDCoV N protein have also proven useful in fluorescent antibody and immunohistochemistry staining methods for identification of PDCoV-infected cells or intestinal tissues [23]. However, the cross-reactivity between porcine coronaviruses in these assays makes accurate diagnoses difficult [24,25,26], thus development of discriminate diagnostic assays for PDCoV is essential. 

In this study, the N protein of PDCoV was expressed in E. coli, purified, then used to produce mouse monoclonal antibodies. The epitope (EP-4E88/309-KPKQQKKPK-317) of the antibody with the highest N protein binding affinity was extensively investigated. Sequence alignment analysis revealed that the sequence of EP-4E88 is highly conserved among porcine deltacoronavirus strains, but has very low sequence similarity to other porcine coronavirus (PEDV, TGEV, PRCV, SADS-CoV, PHEV). Among them, TGEV, PRCV N protein are identical, because PRCV is a S gene deletion of TGEV [27]. Besides, SADS-CoV also known as swine enteric alphacoronavirus (SeACoV) [28] and porcine enteric alphacoronavirus (PEAV) [29]. The results of I-TASSER server for 3D structure prediction showed that amino acids of EP-4E88 was in close proximity and may be exposed on the surface of the N protein. Our findings on this PDCoV N protein epitope added insight for developing epitope-associated diagnostics and vaccines.

## 2. Results

### 2.1. Expression, Purification, and Characterization of Full-Length Porcine Deltacoronavirus (PDCoV) Recombinant N Protein (rNP)

**Figure 1:**
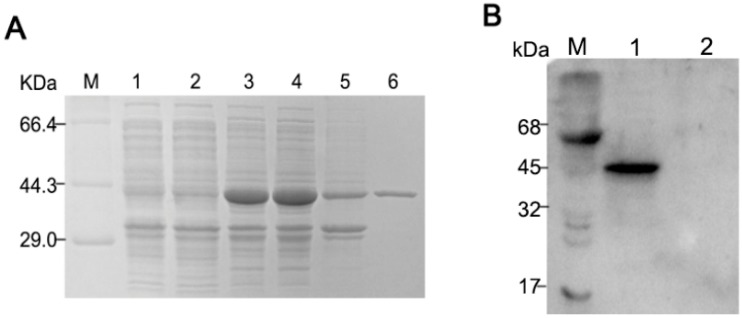
Expression and purification of His-tagged Porcine deltacoronavirus (PDCoV) N-protein. (**A**) SDS-PAGE of pET28a-N transfected BL21 (DE3) cells. M, protein molecular weight marker; lane 1: *E. coli* BL21 with empty vector pET-28a (+); lane 2: Uninduced *E.coli* BL21 with pET-28a-N; lane 3, IPTG-induced pET28a-N transfected cells prior to sonication, lane 4, supernatant of pET28a-N transfected cells post sonication; lane 5: the precipitates of bacterium solution; lane 6: Ni-NTA purified rNP from supernatant; (**B**) Western blot of *E. coli* expressed pET28a-N (lane 1) and empty vector pET-28a (lane 2), probed with hyperimmune pig anti-PDCoV.

As seen in the SDS-PAGE results (Figure 1A), the expressed PDCoV-rNP was soluble and found predominantly in the supernatant post sonication (lane 4), and lanes 6 show the Ni-NTA purified rNP at the expected size, 44 kDa. The Western blot (Figure 1B), demonstrates that the recombinant N protein was specifically recognized by the pig anti-PDCoV hyperimmune serum at a 1:200 dilutions.

### 2.2. Production and Screening of PDCoV rNP mAbs

**Figure 2:**
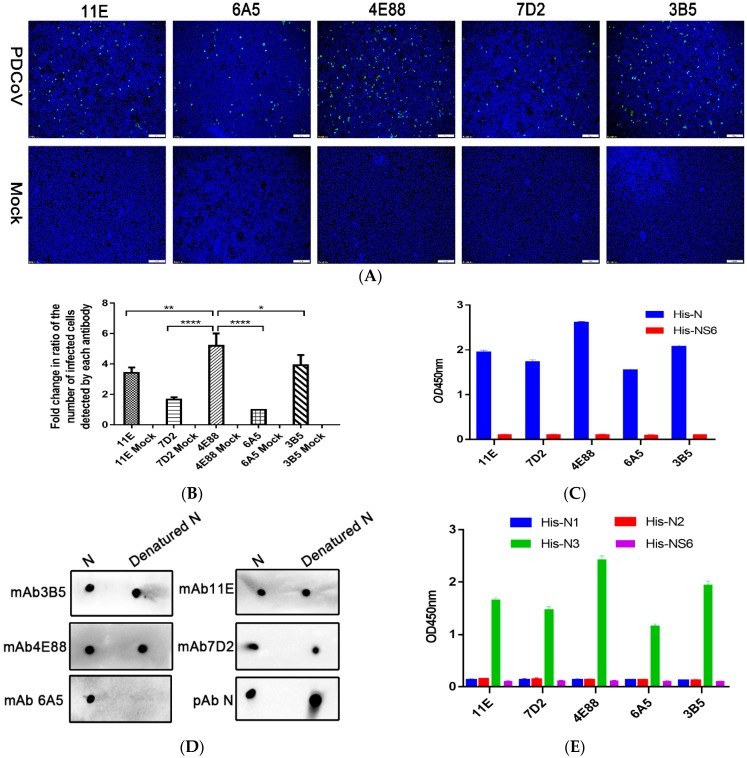
Activity of anti-PDCoV rNP mAbs. (**A**) Immunoinfluscent assay (IFA) of the five mAbs in PDCoV infected cells. Magnification = 10×; (**B**) Quantification of the virus-infected cells detected by each monoclonal shown in panel A. * *p* = 0.0483, ** *p* = 0.0049, **** *p* < 0.0001 for 4E88 compared to 3B5, 11E, 7D2, 6A5, respectively; (**C**) Activity of the antibodies with His-N and His-NS6 by ELISA; (**D**) Linear epitope identification of five monoclonals by dot blot with denatured and native N protein; (**E**) ELISA assay of the reactivity of each monoclonal with the N1,N2, and N3 protein. The experiments were repeated three times.

Purified PDCoV rNP was used to immunize BALB/c mice; the mouse with the highest antibody titer was sacrificed for hybridoma production. Hybridomas were screened four times by indirect ELISA, and ultimately five hybridomas (11E, 7D2, 4E88, 6A5, and 3B5) were chosen for further testing. The hybridomas were identified as IgG1 ĸ light chain isotype using a mouse monoclonal antibody isotyping ELISA kit (Proteintech Group, Inc.). As can be seen from the representative indirect immunoinfluscent assay (IFA) images (Figure 2A) the monoclonal 4E88 showed the greatest reactivity with the N protein in infected cells. The reactivity of infected cells detected by each monoclonal was quantitated by Imagine Pro plus 6.0 and the results are shown in the bar graph in Figure 2B. ELISA results (Figure 2C) also demonstrated that monoclonal 4E88 had greater reactivity with the N-protein than did the other mAbs. PDCoV NS6 was used as a negative control. To determine if the binding of the mAbs to the N protein required its conformational integrity, native and denatured N was dotted onto PVDF membrane and reacted with each mAb. The results (Figure 2D) show that 4E88, 3B5, 11E, and 7D2 react well with denatured N protein, indicating they recognize linear epitopes. Monoclonal 6A5 did not react with denatured N protein, indicating that it may recognize a conformational epitope.

Next, IEDB Analysis Resource online analysis software was used to predict the B-cell epitope of N protein. Based on the analysis results, we divided the N protein into three parts: N1 (1-124aa), N2 (113-240aa), N3 (221-342aa). The ELISA results (Figure 2E), showed that all the five mAbs recognized the N3 fragment but did not react with N1 and N2. 

### 2.3. Epitope Mapping of PDCoV NP

**Figure 3:**
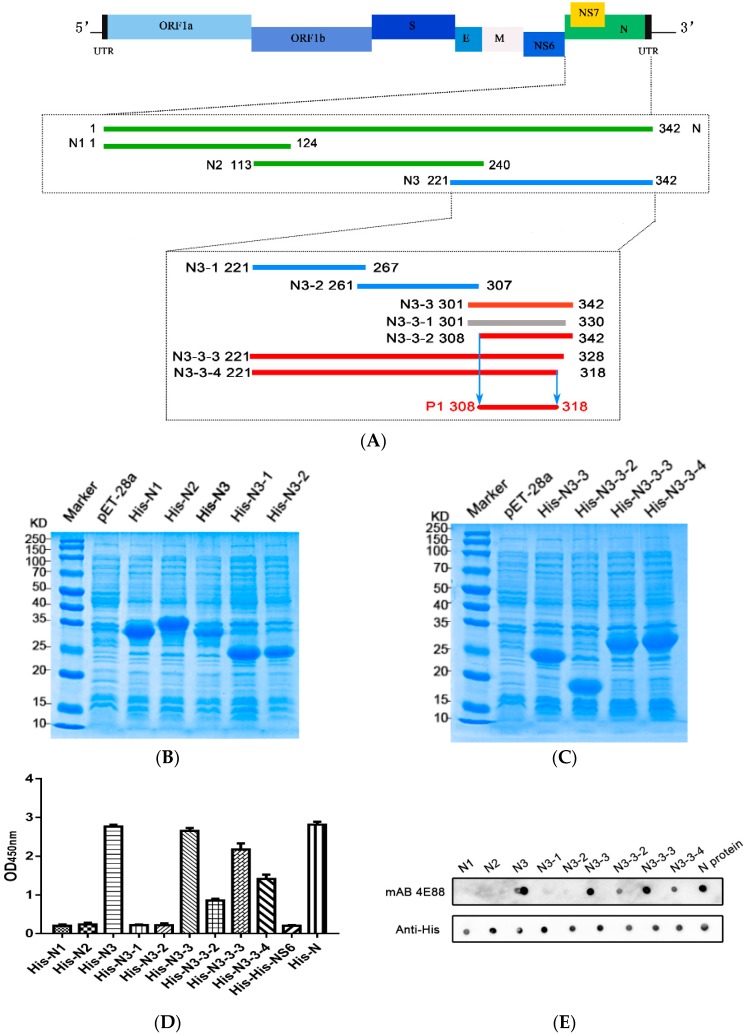
PDCoV N-protein epitope mapping. (**A**) Schematic of PDCoV genome organization and N-protein epitope mapping. The segments recognized by monoclonal 4E88 are represented by red lines, the segments unrecognized by 4E88 are represented by green and blue lines, and the segment that was unsuccessfully expressed is represented by gray; (**B**,**C**) SDS-PAGE of the N-protein truncations expressed in *E. coli*; (**D**,**E**) ELISA and dot blot assays of the reactivity of monoclonal 4E88 with the N-protein truncations. The experiments were performed in triplicate.

**Figure 4:**
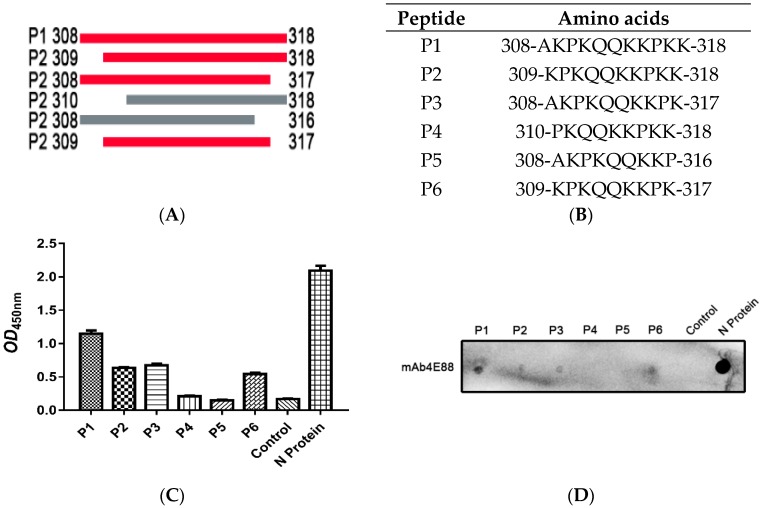
Identification of the minimal 4E88 epitope. Six peptides (P1, P2, P3, P4, P5, P6) were synthesized to detect the minimal epitope (**A**,**B**) and tested for reactivity with 4E88 by ELISA (**C**) and dot blot assays (**D**). The experiments were repeated three times.

To locate the epitope on N3 recognized by mAb 4E88, three truncations of N3 were constructed: N3-1 (221-267 aa), N3-2 (261-307 aa), and N3-3 (301-342 aa) (Figure 3A). ELISA (Figure 3D) and dot blot (Figure 3E) results showed that mAb 4E88 reacted only with the N3-3 fragment. From here, four truncations of the N3-3 fragment were constructed: N3-3-1 (301-330 aa), N3-3-2 (308-342aa), N3-3-3 (221-328 aa), and N3-3-4 (221-318 aa). N3-3-1 was not expressed successfully, and although mAb 4E88 did react with N3-3-2 the signal was weak. Both N3-3-3 and N3-3-4, which contain most of the N3-3-1 region, reacted with mAb 4E88, but the N3-3-3 region reacted strongly while N3-3-4 reacted somewhat weakly. These results demonstrated that the amino acids 308-318 are at a minimum necessary for the 4E88 interaction. Peptides spanning these amino acids were synthesized (Sangon Biotech, China) (Figure 4A,B) for subsequent experiments.

### 2.4. Identification of the Minimal Epitope

The synthesized peptides were used as antigens in ELISA (Figure 4C) and dot blot assays (Figure 4D), the results showed that mAb 4E88 reacted most strongly with P1, reacted about half as well with P2, P3, and P6, and did not react with P4 or P5, demonstrating that the 4E88 minimal epitope is amino acids 309-317. 

### 2.5. Cross-Reactivity Analysis 

**Figure 5:**
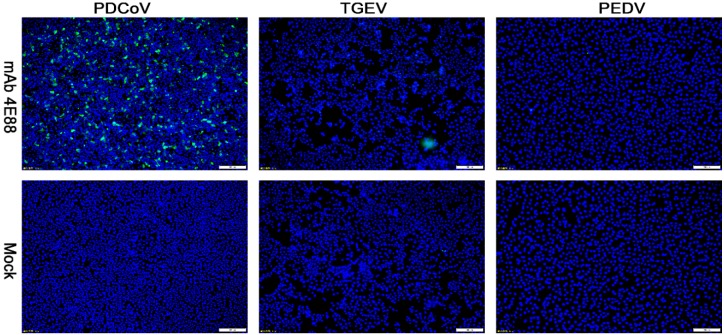
Reactivity of mAb 4E88 with PDCoV, TGEV, and PEDV-infected cells determined by IFA. Binding was visualized with FITC labeled goat anti-mouse antibody, while DAPI was used to visualize the cell nuclei. The strain used for cell infection is shown at the top, and the antibody used for the assay are indicated on the left. Magnification = 10×.

IFA was performed to investigate whether PDCoV cross-reacts with PEDV and TGEV on the epitope of EP-4E88 (aa 309-KPKQQKKPK-317). The mAb 4E88 was used as primary antibody. Figure 5 shows that no fluorescent signals were observed in TGEV infected ST cells and PEDV-infected Vero cells. This result shows that PDCoV EP-4E88 does not cross-react with TGEV and PEDV.

### 2.6. Homology Analysis 

**Figure 6:**
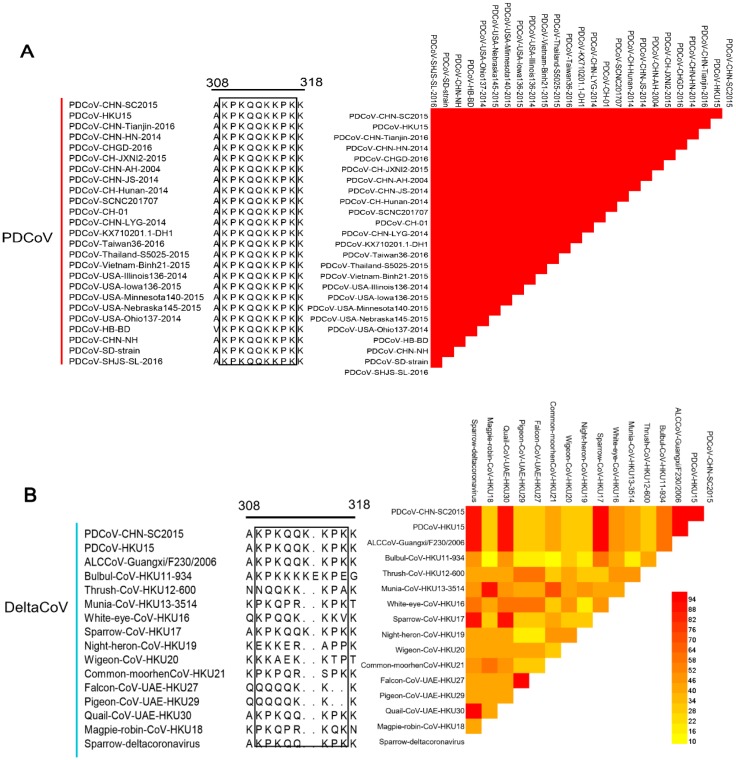

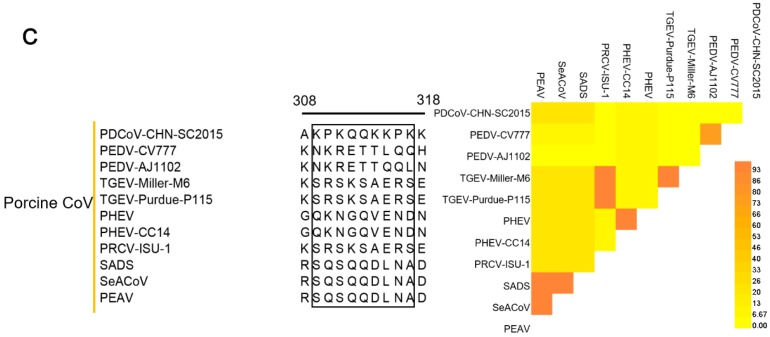
Comparison of the 4E88 epitope amino acid sequence among different virus strains. (**A**) EP-4E88 sequences from strains of PDCoV. Heat map of homology comparison between PDCoV CHN-SC2015 strain and other 24 PDCoV reference strains in GenBank. The sequences of EP-4E88 for all strains are surrounded by black frames; (**B**) EP-4E88 sequences from strains of other 15 deltacoronaviruses. Heat map of homology comparison between PDCoV CHN-SC2015 strain and 15 deltacoronavirus strains in GenBank; (**C**) EP-4E88 sequences from strains of other 11 porcine coronavirus. Heat map of homology comparison between PDCoV CHN-SC2015 strain and 11 porcine coronavirus reference strains in GenBank. The level of homology were analyzed by MAFFT v7.037 and analyzed by DNASTAR.

To explore the level of conservation of the 4E88 epitope, 25 PDCoV strains from GenBank were selected for sequence alignment (Figure 6A), which was done using DNA Star. The results show that the EP-4E88 (aa 309-KPKQQKKPK-317) is highly conserved among the PDCoV strains analyzed, with shared sequence similarity of 100% (Figure 6A). Sixteen strains in the genus *Deltacoronavirus* were chosen for further sequence alignment. The results revealed that PDCoV, sparrow coronavirus (HKU17) and Asian leopard cat coronavirus (ALCCoV) share 100% sequence similarity in the position of epitope 4E88 (Figure 6B). The newly discovered Quail deltacoronavirus (UAE-HKU30) and Sparrow deltacoronavirus (SpDCoV) share 90% sequence similarity with PDCoV in epitope 4E88, having one amino acid deletion at lys^314^. The other deltacoronavirus have 30–60% sequence similarity with PDCoV in epitope 4E88. 

PDCoV is the only porcine deltacoronavirus; sequence alignment of the 4E88 epitope with other porcine coronaviruses was performed to determine conservation among coronavirus genera. The results showed very low sequence similarity, the highest being 22.2% (Figure 6C). 

### 2.7. Distructure of EP-4E88

**Figure 7:**
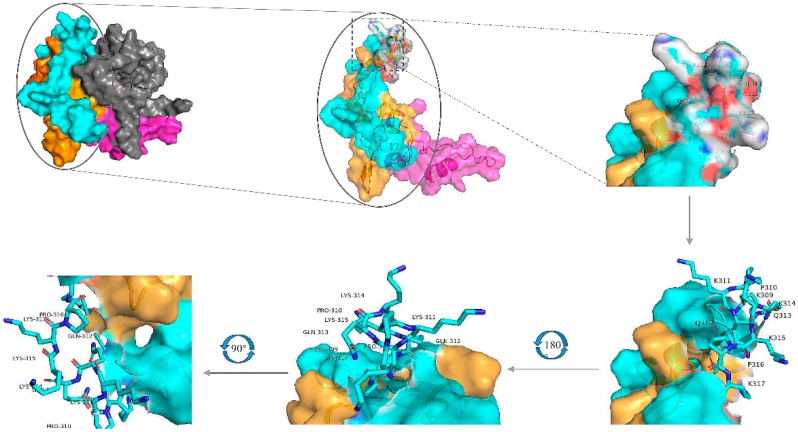
Model of the I-TASSER predicted 3D structure of the N-protein, visualized using the PyMOL molecular graphics and modeling system. The overall structure is shown on the upper left, the yellow areas represent aa231-291, blue areas represent aa292-342, grey areas represent aa1-171, and pink areas represent aa172-230. The EP-4E88 residues (aa 309-KPKQQKKPK-317) are shown as a stick figure and are displayed at different angles of rotation.

A 3-dimensional model of the PDCoV N-protein was constructed based on seven templates in the I-TASSER server website(Figure 7). Top five final models predicted by I-TASSER and its C-score. In I-TASSER, C-score has the function of estimating the quality of predicted models. Our models had C-scores in the range of [−4.26, −3.58], which is nomal for predicted models. The amino acids of EP-4E88 are located in close proximity to one another and are predicted to be exposed on the surface of the N protein, suggesting that EP-4E88 is highly likely to be a linear epitope. 

## 3. Discussion

PDCoV, a novel swine enteropathogen, has spread quickly since first discovered in 2012 in Hong Kong. Its distribution is now nearly worldwide and is causing increasing economic losses to commercial pig industries. Co-infections of PDCoV and other enteric viruses complicates diagnosis and leads to increased morbidity and mortality [12,30]. A discriminate diagnostic tool would be invaluable for efforts in the control of PDCoV and similar viruses. 

Coronavirus nucleocapsid protein is one of the most abundant viral proteins and is the major antigen recognized by convalescent antisera [31]. Compared with other coronavirus proteins, the N protein is a preferred candidate for serological diagnostics because it is abundant and highly conserved among coronavirus species [26]. Leung et al. found [32] that in patients with SARS, IgG most frequently dominated the antibody response and predominantly targeted the viral nucleocapsid. With respect to PDCoV, the S and N proteins have frequently been the focus for the development of diagnostics [15]. The CoV spike protein however, varies due to mutation to a much greater extent that does the N protein, the result of this variation is a lack of diagnostic accuracy [33].

In this study, monoclonal antibodies were produced against purified recombinant N-protein and their epitopes investigated. The five antibodies we tested, all recognized the 122 C-terminal amino acids of the N-protein (aa 221-341), indicating that this section contains the main antigenic epitope of N protein. IEDB (Immune Epitope Database and Analysis Resource) prediction analysis software (http://www.iedb.org/) also proved our point, the results indicated that three epitopes located at the C-terminal of the N-protein sequence, and the largest of 10 predicted epitopes is in amino acids 221-342, this fragment we termed N3. B-cell epitopes are classified as linear or conformational, though it has been reported that 90% of B cell-recognizing epitopes are conformational epitopes, this is due to most of the B-cells epitopes can form conformation in the three dimensional structure, the antigen internalizing process and antigen recognizing ability [34,35,36]. To determine whether our monoclonals recognized conformational epitopes, the N protein was denatured and then tested for reactivity with the mAbs by dot-blot. One mAb (6A5) did not react with the denatured N protein, so we speculated that it recognizes a conformational epitope, the remaining mAbs did react with the denatured N protein so we speculated that these recognize linear epitopes. One mAb (4E88) from our panel was selected for further study of the antigenic epitope of N protein; we chose this antibody because it had the highest reactivity with N-protein in ELISA and dot blot, as well as in PDCoV-infected cells. Moreover, the dominant epitope of the N protein has a greater potential use for early diagnostics than the others.

Identification of PDCoV B-cell epitopes is fundamental for the development of epitope-based diagnostic tools, vaccines, and therapeutic antibodies. Approaches for epitope identification are structural and functional. Structural methods include X-ray crystallography, which can precisely locate the epitope position but it is limited to small soluble proteins, the method is also time consuming and costly, together these impede its widespread use [37]. Functional methods are used to detect the binding activity of antibody with antigen fragments, synthetic peptides or recombinant antigens, and have the advantage of being simpler to conduct [37]. Here, the epitope region recognized by 4E88 (308-AKPKQQKKPKK-318 aa) was determined by serially truncating the N3 portion of the N-protein. From there a series of peptides were synthesized and used as antigen in ELISA and dot blot assays, thus we determined that 309-KPKQQKKPK-317 was the minimal epitope.

Woo et al. [1] found that avian and mammalian deltacoronaviruses share similar genome characteristics and structure, and that avian coronaviruses are the gene source of gamma- and deltacoronaviruses. In our study alignment analysis revealed that the sequence of epitope 4E88 is highly conserved among PDCoV strains; the sequence similarity was also extremely high with Sparrow coronavirus (HKU17), Asian leopard cat coronavirus (ALCCoV), Quail coronavirus (UAE-HKU30), and Sparrow deltacoronavirus (SpDCoV). PDCoV may come from a host jumps event between mammals and birds [38], also supporting this view is genome analysis showing that PDCoV shares a close relationship with HKU17 (96.8% of sequence identity in N), HKU30 (90.9% of sequence identity in N), and SpDCoV (95.3% of sequence identity in N) [1,39]. The shared sequence at position 309-317aa (epitope 4E88) among PDCoV, HKU17, ALCCoV, HKU30, and SpDCoV may contribute to the understanding of the evolutionary relationship between birds and mammals. Similar phenomenon was also found in other virus. Li et al. [40] found a shared epitope, EXE/DPPFG, among six flaviviruses and verified the cross-reactivity by positive sera detection. Chen et al. [41] found that PDCoV N gene-based PCR cross-reacts with SpDCoV because of their relatively conserved regions. The relationship between PDCoV and other animal-originated deltacoronavirus needs further exploration and whether PDCoV EP-4E88 cross-reacts with HKU17, ALCCoV, HKU30, or SpDCoV should be analyzed using two-way serum cross-reactivity.

To date, six porcine coronavirus diseases (PEDV, TGEV, PRCV, SADS-CoV, PHEV, PDCoV) have been reported [42,43]. Because they present with similar clinical symptoms, diagnosis is a challenge, a sensitive and discriminate serologic method is clearly needed. The PDCoV epitope, 4E88 (aa 309-KPKQQKKPK-317), found in this study shares very low sequence similarity (<22.2%) with other swine coronavirus (Figure 6C), such as PEDV (aa 309-NKRETTLQQ-317) and TGEV (aa 309-SRSKSAERS-317), PHEV (aa 309-QKNGQVEND-317), PRCV (aa 309-SRSKSAERS-317), SADS-CoV (aa 309-SQSQDLNA-317) indicating the EP-4E88 is unique to PDCoV and could be used to distinguish PDCoV and from other swine coronaviruses. Cross-reactivity among swine coronaviruses has been reported and is usually associated with the epitope of N protein [24,26,44]. Lin et al. [24] observed that one-way cross-reaction between TGEV Miller hyperimmune pig antisera and various PEDV strains with the TGEV Miller N protein mAb (14G9.3C) was based on an N protein epitope. Xie et al. [26] found two N-terminal epitopes (58-RWRMRRGERIE-68 and 78-LGTGPHAD-85) of the PEDV N protein contribute to the cross-reaction between PEDV and TGEV. Ma et al. [44] found that N protein is responsible for two-way cross-reactivity between PEDV and PDCoV; four regions (47-GYW-49, 67-FYYTGTGPRGNLKY-82, 194-PKG-197, and 329-EWD-332) are highly conserved and likely account for the cross-reaction [44]. 

To our knowledge, this is the first report to identify the PDCoV N-protein antigenic epitope EP-4E88 (aa 309-NKRETTLQQ-317). It is a linear B-cell epitope and highly conserved among PDCoV strains and other porcine coronaviruses, EP-4E88 also shares high sequence identity with four non-swine deltacoronavirus. Our findings provide valuable insight into the evolutionary and serological relationship among PDCoV and non-swine deltacoronavirus, and for the development of PDCoV epitope-associated diagnostics and vaccine design.

## 4. Materials and Methods

### 4.1. Ethics Statement

All animal experiments were approved by the Institutional Animal Care and Use Committee of Sichuan Agricultural University (IACUC#RW2016-090, approval date: 8 September 2016), and were performed in strict accordance with the Care and Use of Laboratory Animals guidelines and regulations of the Ministry of Science and Technology of the People’s Republic of China. 

### 4.2. Virus and Cells

ST cells (ATCC CRL-1746), Sp2/0-Ag14 (ATCC CRL-1581) cells and Vero cells (ATCC CCL-81) were maintained at 37 °C in a humidified 5% CO_2_ atmosphere in Dulbecco’s modified Eagle medium (DMEM; Gibco, Carlsbad, CA, USA) supplemented with 10% heat-inactivated fetal bovine serum (PAN-Biotech, Aigenbach, Germany) and 1% antibiotic-antimycotic (Solarbio, Beijing, China). The PDCoV strain CHN-SC2015 (GenBank accession No.MK355396), PEDV-CV777 strain (GenBank accession No. AF353511.1), TGEV-H strain was preserved by the Laboratory of Research Center of Swine Disease in Sichuan Agricultural University. For PDCoV propagation, ST cells at 90% confluence were washed three times with DMEM supplemented with 5 μg/mL trypsin-EDTA (maintenance medium) and then inoculated with 1 mL of PDCoV. The virus was removed and 6 mL of maintenance medium was added. When CPE was observed, generally at 2 days post-infection, the cells were harvested then stored at −80 °C until further use. The titers of CHN-SC2015 obtained were up to 10^6.64^ TCID50/mL. For PEDV propagation, Vero cells at 90% confluence were washed three times with DMEM supplemented with 10 μg/mL trypsin-EDTA (maintenance medium) and then inoculated with 1 mL of PEDV. When CPE was observed, generally at 3 days post-infection, the cells were harvested then stored at −80 °C until further use. For TGEV propagation, ST cells at 90% confluence were washed three times with DMEM supplemented with 5 μg/mL trypsin-EDTA (maintenance medium) and then inoculated with 1 mL of TGEV. When CPE was observed, generally at 2 days post-infection, the cells were harvested then stored at −80 °C until further use. 

### 4.3. Construction of Full-Length and Truncated Recombinant N-Protein

PDCoV CHN-SC2015 genomic RNA was extracted using TRIzol Reagent (Sangon Biotech, Shanghai, China) according to the manufacturer’s instructions. The N gene was amplified by RT-PCR with primers (Table 1) designed with Primer 5.0. The N gene amplicon (1029 bp) was inserted into the pET-28a (+) expression vector (Novagen, Madison, WI, USA) between the EcoR I and Xho I sites. The integrity of the resulting construct (pET28-N) was verified by restriction enzyme digestion, PCR, and DNA sequencing. pET28-N was transformed into Transetta (DE3) *Escherichia coli*. Protein expression was induced with 0.8 mM isopropyl-β-galactopyranoside (IPTG) for 3 h at 30 °C. Bacteria were collected by centrifugation and lysed by ultrasonication, then centrifuged again; the recombinant N protein (rNP) was purified from the supernatant post sonication by Ni-NTA His-Bind Resin (Bio-Rad, Hercules, CA, USA) according to the instructions. The concentration of the purified rNP was analyzed by SDS-PAGE. Using the same methodology, a series of truncated N-proteins were produced: His-N1, His-N2, His-N3, His-N3-1, His-N3-2, His-N3-3, His-N3-3-1, His-N3-3-2, His-N3-3-3, His-N3-3-4 (Figure 3A).

### 4.4. Western Blotting

Western blotting was used to test the reactivity of hyperimmune pig anti-serum against the recombinant N protein. Purified N protein and pET-28a (+) empty vector were subjected to SDS-PAGE then transferred to a PVDF membrane. The membrane was blocked with 5% skim milk in PBST (PBS/0.05% Tween-20) for 1.5 h then incubated with anti-PDCoV pig hyperimmune serum (1:200) overnight at 4 °C overnight. The membrane was washed three times with PBST then incubated with HRP-goat anti-pig IgG (1:5000) for 1 h at 37 °C. The membrane was washed four times with PBST and the proteins were visualized using enhanced chemiluminescence reagents (ECL; Bio-Rad, Hercules, CA, USA).

### 4.5. Production of Anti-rNP mAbs

Six-week-old female BALB/c mice purchased from Chengdu Dossy Experimental Animal Co, Ltd., were inoculated via subcutaneous injection with purified rN protein (100 μg/mouse) mixed with an equal volume of Montanide Gel 01 PR adjuvant (Montanide, SEPPIC, Puteaux, France). Mice were boosted twice, at 2-week intervals, with the same immunogen and adjuvant. Pre-immune serum samples were taken from all mice and tested for reactivity against purified rNP by indirect ELISA. Two weeks after the final boost, mice were sacrificed and spleens removed. Spleen cells were fused with Sp2/0 Ag14 cells, and hybridomas were selected in HAT and HT medium. Culture supernatants from individual hybridoma clones were screened for reactivity with rNP by indirect ELISA. Reactive hybridomas were subcloning three times by limiting dilution, stable hybridomas were injected into the abdominal cavity of sensitized mice to produce ascites. Antibodies were purified twice, first using the octylic acid ammonium sulfate method (CA-AS), then by HPLC through a diethylaminoethyl column. The identification of isotype in prepared mAbs by mouse monoclonal antibody isotyping ELISA kit (Proteintech Group, Inc., Wuhan, China).

### 4.6. Denatured Protein 

To identify mAbs that recognize conformational or linear epitopes, the N3 proteins were denatured as previously described [34]. Briefly, the purified rN proteins were mixed with 6× protein-loading buffer with DTT (TransGen Biotech, Beijing, China) then heated for 8 min at 95 °C; this procedure fully denatures secondary structure. The denatured proteins were tested with the mAbs using a dot-blot assay. 

### 4.7. IFA

ST cells were grown in 12-well plates until 80% confluent. Half the wells were infected with PDCoV (MOI = 0.1) and half were mock infected. After 36 h post-infection, cells were then washed twice with PBS, fixed with 4% formaldehyde in PBS for 30 min, then permeabilized with 0.5% Triton-X-100 for 30 min at room temperature. Cells were washed again and blocked with 2% BSA in PBS for 1.5 h then incubated with each of the five anti-N mAb for 1 h in PBS with 1% BSA. Cells were washed three times and incubated with FITC labeled goat anti-rabbit IgG (1:500 in PBST). Nuclei were stained with DAPI (Solarbio, Beijing, China). The Image-Pro Plus 6.0 was used to analyze number of immunfluoresnce from six different stained images. The FITC-positive cells of mAb 6A5 was set to one-fold.

### 4.8. ELISA 

Indirect ELISA was performed as described in Guo et al. 2006 [45]. Briefly, the wells of a 96-well ELISA plate were coated with 1 μg/well of protein overnight at 4 °C. The wells were rinsed, then blocked with 5% non-fat dry milk in TBST for 1 h at room temperature. Wells were rinsed three times with TBST then incubated with mAb (1:500) for 1 h at 37 °C. Wells were rinsed again and incubated with HRP-conjugated goat anti-mouse IgG, (1:5000) for 1 h at 37 °C. After final rinsing wells were incubated with tetramethylbenzidine (TMB) for 15 min, color development was stopped with 3M H_2_SO_4_. The *OD*_450_ was read by ELISA plate reader (Bio-Rad, Hercules, CA, USA).

### 4.9. Dot-Blot Analysis

Dot-blot hybridization for identification of linear epitopes was based on the method described by Chen et al. 2017 [46]. Briefly, PVDF membranes were soaked in dimethyl sulfoxide then in methanol. Total of 1 μL (0.5 μg) of protein was spotted onto the treated membrane, then air-dried for 10 min. The membrane was blocked with 2% BSA in TBST for 30 min at room temperature, then incubated with mAb (1:200) in 2% BSA/TBST for 1 h at 37 °C. The membrane was rinsed then incubated with goat anti-mouse IgG (1:5000) for 1 h at 37 °C. The signal was developed using enhanced chemiluminescence reagents (ECL; Bio-Rad, Hercules, CA, USA). 

### 4.10. Identification of the Minimal 4E88 Epitope

To determine the minimal B-cell epitope recognized by mAb 4E88, five peptides spanning various lengths of aa308-318 were commercially synthesized (Sangon Biotech, Shanghai, China). The amino acid sequence of these peptides (P1–P6) are shown in Figure 3A,B. The reactivity of 4E88 with the peptides was tested by dot-blotting and ELISA as previous described [47]. Specially, peptides were dissolved in DMSO (Solarbio) to a concentration of 10 mg/mL, for dot-blotting 1 μL (10 μg) was spotted onto PVDF membrane, and for ELISA, 100 μL (1 mg) were aliquoted into wells and incubated at 4 °C for 15 h. 

### 4.11. Sequence Homology

To determine whether the epitope recognized by mAb 4E88 was conserved among PDCoV strains, the amino acid sequences of 25 PDCoV strains in GenBank (Table 2) were aligned with the sequence in EP-4E88 using MAFFT v7.037 and analyzed by DNASTAR. The EP-4E88 sequence was also aligned with other deltacoronavirus and porcine coronavirus (Table 3 and Table 4) as described. The results were rendered in figure form with ESPript 3.

### 4.12. Three-Dimensional Structure Prediction

I-TASSER (https://zhanglab.ccmb.med.umich.edu/I-TASSER/) was used to predict the three-dimensional structure of the full-length PDCoV N-protein as described [48,49,50]. Seven threading templates in Protein Data Bank (PDB) were selected for construction by this program (2gecB, 5gaoE, 1sskA, 4n16A, 4j3kA, 1ssk, 2gecA). Figures were generated using the PyMOL molecular visualization system.

### 4.13. Statistical Analysis

The experiments were repeated three times. All statistical data were analyzed by GraphPad Prism version 7.0 and expressed as mean ± SD. The differences among the five monoclonals were analyzed using the one-way ANOVA. Statistical changes marked by * *p* value < 0.05, ** *p* value < 0.01, *** *p* value < 0.001, **** *p* value < 0.0001.

## 5. Conclusions

In this study, five monoclonals of PDCoV N protein were produced. Of the five monoclonals, mAB 4E88 had highest binding affinity with the N protein and was chosen for identifying epitope. EP-4E88 (aa309-KPKQQKKPK-317) was the minimal epitope which recognized by mAb 4E88. Homology analysis showed that the EP-4E88 sequence is highly conserved among PDCoV strains and several deltacoronavirus but shared very low similarity with other porcine coronaviruses. Therefore, EP-4E88 lays a good foundation for applied research associated with PDCoV diagnosis.

## Figures and Tables

**Table 1:** Sequence of the oligonucleotides used for PCR.

Segment		Sequences (5′-3′)	Positions (Amino Acids)
N1	N1-F	CGGGATCC ATGGCCGCACCAGTAGTC	1-124
	N1-R	*CCCTCGAG* TAGCAGCTGATGTTTAGGATT	
N2	N2-F	CGGGATCC TCGGGAGCTGACACTTCTATTA	113-240
	N2-R	*CCCTCGAG* TGCCCCTGCCTGAAAGTTG	
N3	N3-F	CGGGATCC AAGACGGGTATGGCTGATCC	221-342
	N3-R	*CCCTCGAG* CTACGCTGCTGATTCCTGCT	
N3-1	N3-1-F	CGGGATCC TCTCGTACTGGTGCCAATGTCG	221-267
	N3-1-R	*CCCTCGAG* GAGCGCATCCTTAAGTCTCTCATAG	
N3-2	N3-2-F	CGGGATCC TTCTCTTACTCAATCACAGTCAAGG	261-307
	N3-2-R	*CCCTCGAG* GACTGGTCTTGTTTGTCAGGCTT	
N3-3	N3-3-F	CGGGATCC CCTGACAAACAAGACCAGTCTG	301-342
	N3-3-R	*CCCTCGAG* CGCTGCTGATTCCTGCTTTA	
N3-3-1	N3-3-1-F	CGGGATCC CCTGACAAACAAGACCAGTCTGCTA	301-330
	N3-3-1-R	*CCCTCGAG* CCACTCCCAATCCTGTTTGTCTG	
N3-3-2	N3-3-2-F	CGGGATCC GCTAAACCCAAACAGCAGAAGAAAC	308-342
	N3-3-2-R	*CCCTCGAG* CGCTGCTGATTCCTGCTTTAT	
N3-3-3	N3-3-3-F	CGGGATCC AAGACGGGTATGGCTGATCC	221-328
	N3-3-3-R	*CCCTCGAG* CCAATCCTGTTTGTCTGCTG	
N3-3-4	N3-3-4-F	CGGGATCC AAGACGGGTATGGCTGATCC	221-318
	N3-3-4-R	*CCCTCGAG* CTTTTTAGGTTTCTTCTGCTGTTTG	

Restriction endonuclease sites: BamHI (underlined) and XhoI (italic).

**Table 2:** PDCoV strains used to align the sequences of EP-4E88.

Strains	Country	Collection Date	Accession Number	Lengths of N-Protein (Amino Acid)
PDCoV-CHN-SC2015	China	2015	QDH76192.1	342
PDCoV-CHN-JXJGS01-2016	China	2016	ASK86338.1	342
PDCoV-CHN-Tianjin-2016	China	2016	APG38202.1	342
PDCoV-CHN-HN-2014	China	2014	ALS54090.1	342
PDCoV-CHGD-2016	China	2016	AYU65238.1	342
PDCoV-CH-JXNI2-2015	China	2015	ALA13749.1	342
PDCoV-CHN-AH-2004	China	2004	AKC54432.1	342
PDCoV-CHN-JS-2014	China	2014	AKC54446.1	342
PDCoV-CH-Hunan-2014	China	2014	AUG59160.1	342
PDCoV-SCNC201707	China	2017	AZL30771.1	342
PDCoV-CH-01	China	2016	AQS99157.1	342
PDCoV-CHN-LYG-2014	China	2014	AML83920.1	342
PDCoV-KX710201.1-DH1	China	2016	ASW22235.1	342
PDCoV-Taiwan36-2016	China	2016	KY586149.1	342
PDCoV-Thailand-S5025-2015	Thailand	2015	KU051656.1	342
PDCoV-Vietnam-Binh21-2015	Vietnam	2015	APZ76702.1	342
PDCoV-USA-Illinois136-2014	USA	2014	AIB07804.1	342
PDCoV-USA-Iowa136-2015	USA	2015	ANI85829.1	342
PDCoV-USA-Minnesota140-2015	USA	2015	ANI85836.1	342
PDCoV-USA-Nebraska145-2015	USA	2015	ANI85850.1	342
PDCoV-USA-Ohio137-2014	USA	2014	KJ601780.1	342
PDCoV-HB-BD	China	2017	ATJ00133.1	342
PDCoV-CHN-NH	China	2015	ANA78447.1	342
PDCoV-SD-strain	China	2014	ASR75150.1	342
PDCoV-SHJS-SL-2016	China	2016	AUH28254.1	342

**Table 3:** Deltacoronavirus strains used for EP-4E88 sequence alignment.

Strains	Country	Collection Date	Accession Number	Lengths of N-Protein (Amino Acid)
PDCoV-HKU15	China	2009	YP_005352835.1	342
Asian leopard cat coronavirus (ALCCoV) Guangxi/F230/2006	China	2006	ABQ39962.1	342
Bulbul-CoV-HKU11-934	China	2007	ACJ12039.1	349
Thrush-CoV-HKU12-600	China	2007	ACJ12057.1	343
Munia-CoV-HKU13-3514	China	2007	ACJ12066.1	352
White-eye-CoV-HKU16	China	2007	YP-005352842.1	347
Sparrow-CoV-HKU17	China	2007	YP-005352850.1	342
Night-heron-CoV-HKU19	China	2007	YP-005352867.1	342
Wigeon-CoV-HKU20	China	2008	YP-005352875.1	350
Common-moorhen CoV-HKU21	China	2007	YP-005352885.1	351
Falcon-CoV-UAE-HKU27	China	2013	BBC54826.1	344
Pigeon-CoV-UAE-HKU29	China	2017	BBC54846.1	344
Quail-CoV-UAE-HKU30	China	2017	BBC54865.1	341
Magpie-robin-CoV-HKU18	China	2007	YP-005352858.1	346
Sparrow-deltacoronavirus	USA	2017	AWV67111.1	341

**Table 4:** Porcine coronavirus strains used for EP-4E88 sequence alignment.

Strains	Country	Collection Date	Accession Number	Lengths of N-Protein (Amino Acid)
PEDV-CV777	Belgium	1977	AF353511	441
PEDV-AJ1102	China	2011	JX188454	441
TGEV-Purdue P115	USA	2006	DQ811788	382
TGEV-Miller M6	USA	2006	DQ811785	382
PRCV-ISU-1	USA	2006	DQ811787	382
PHEV-VW572	Belgium	2005	DQ011855	449
PHEV-CC14	China	2014	MF083115	449
SADS-CoV/CN/GDWT/2017	China	2017	MG557844	375
PEAV-GDS04	China	2017	MH697599	375
SeACoV-p10	China	2018	MK977618	375
